# Phloretin Ameliorates Testosterone-Induced Benign Prostatic Hyperplasia in Rats by Regulating the Inflammatory Response, Oxidative Stress and Apoptosis

**DOI:** 10.3390/life11080743

**Published:** 2021-07-26

**Authors:** Chao Yu Hsu, Yi Sheng Lin, Wei Chun Weng, Lauren Panny, Hsiang Lai Chen, Min Che Tung, Yen Chuan Ou, Chi Chien Lin, Che Hsueh Yang

**Affiliations:** 1 Division of Urology, Department of Surgery, Tungs’ Taichung MetroHarbor Hospital, Taichung 435, Taiwan; t4361@ms.sltung.com.tw (C.Y.H.); t12197@ms.sltung.com.tw (Y.S.L.); t10527@ms.sltung.com.tw (W.C.W.); t3811@ms.sltung.com.tw (H.L.C.); t1142@ms.sltung.com.tw (M.C.T.); 2 PhD Program in Translational Medicine, Rong Hsing Research Center for Transitional Medicine, National Chung Hsing University, Taichung 402, Taiwan; 3 Department of Nursing, Jen-Teh Junior College of Medicine, Nursing and Management, Miaoli 356, Taiwan; 4 Department of Biomedical Sciences and Pathobiology, Virginia-Maryland College of Veterinary Medicine, Virginia Polytechnic Institute and State University, Blacksburg, VA 24061, USA; laurenpanny@vt.edu; 5 Institute of Biomedical Science, The iEGG and Animal Biotechnology Center, National Chung-Hsing University, Taichung 402, Taiwan; 6 Department of Biotechnology, Asia University, Taichung 413, Taiwan; 7 Department of Medical Research, China Medical University Hospital, China Medical University, Taichung 406, Taiwan; 8 Department of Medical Research, Taichung Veterans General Hospital, Taichung 407, Taiwan; 9 Department of Pharmacology, College of Medicine, Kaohsiung Medical University, Kaohsiung 807, Taiwan

**Keywords:** prostatic hyperplasia, phloretin, anti-inflammatory, antioxidants, apoptosis

## Abstract

The inflammatory process is proposed to be one of the factors to benign prostatic enlargement (BPH), and this is the first study examining the anti-inflammatory ability of phloretin in treating rats with testosterone-induced BPH. BPH would be induced by testosterone (10 mg/kg/day testosterone subcutaneously for 28 days), and the other groups of rats were treated with phloretin 50 mg/kg/day or 100 mg/kg/day orally (phr50 or phr100 group) after induction. Prostate weight and prostate weight to body weight ratio were significantly reduced in the Phr100 group. Reduced dihydrotestosterone without interfering with 5α-reductase was observed in the phr100 group. In inflammatory proteins, reduced IL-6, IL-8, IL-17, NF-κB, and COX-2 were seen in the phr100 group. In reactive oxygen species, malondialdehyde was reduced, and superoxide dismutase and glutathione peroxidase were elevated in the phr100 group. In apoptotic assessment, elevated cleaved caspase-3 was observed in rats of the phr100 group. Enhanced pro-apoptotic Bax and reduced anti-apoptotic Bc1-2 could be seen in the phr100 group. In histological stains, markedly decreased glandular hyperplasia and proliferative cell nuclear antigen were observed with reduced expression in the phr100 group. Meanwhile, positive cells of terminal deoxynucleotidyl transferase dUTP nick end labeling were increased in the phr100 group. In conclusion, the treatment of phloretin 100 mg/kg/day could ameliorate testosterone-induced BPH.

## 1. Introduction

Prostatic enlargement (PE), which is clinically diagnosed when the volume of the prostate is >30 mL on ultrasound and features hyperplasia in both stromal and epithelial cells in the prostate. It is mostly caused by an adenoma, called benign prostatic hyperplasia (BPH), in different zones and can lead to an elevated detrusor muscle pressure and decreased micturition flow rate in urodynamic, leading to bladder outlet obstruction (BOO). BPH is most frequently diagnosed at the transition zone of the prostate, and BOO may become worse if the PE protrudes into the bladder or if the median lobe is extremely enlarged. BOO, which can be either storage or voiding symptoms, is the most common problem for men with PE seeking medical help and can lead to costly medical expenses. According to the literature [1], most urologists and patients start treatment with medicines of alpha-blockers or 5-α reductase (5α-R) inhibitors, with the most frequently prescribed drug being alpha-blockers [1]. Although alpha-blockers are more efficient in improving the maximal flow rate than in combination with a 5α-R inhibitor, it is insufficient in controlling the International Prostate Symptom Score, prostatic volume, and transitional zone volume [2]. However, a combination of an alpha-blocker and 5α-R inhibitor would result in more significant adverse effects such as problems in ejaculation, erection, and libido [2]. These adverse effects would compromise men’s quality of life and decrease adherence to therapies.

Epidemiologically, the prevalence of PE and BPH increases with aging. Evidence from autopsies has demonstrated that the prevalence of BPH was >50% and >80% in men >60 and >70 years old, respectively [3]. Androgens have an essential role in BPH. Testosterone is transformed into its active metabolite dihydrotestosterone (DHT) by type-2 5α-R in the stromal and epithelial cells of the prostate. It then stimulates cell growth via autocrine or paracrine signal [4]. Aside from the influence of androgens, inflammation has emerged as a factor that can affect the development of BPH [5], with a three-hit process being proposed. After an initial infectious episode caused by either bacteria or viruses (the first hit), inflammation would be sustained by abnormal metabolic conditions (the second hit), particularly hypercholesterolemia, and eventually be maintained by hypogonadism and/or hyperestrogenism (the third hit), leading to overexpressive Lectin-like oxidized low-density lipoprotein receptor-1 (LOX-1) and reactive prostatic cell. A complete or partial of this three-hit process would result in the overexpression of the toll-like receptors (TLR4) in the prostatic cells, which would then act as antigen-presenting cells (APC) and stimulate the human prostate-associated lymphoid tissue (PALT). The final stimulus of PALT, made up of T and B lymphocytes and existing in the periglandular stroma, would eventually lead to the excessive production of growth factors, promoting hyperplasia. According to a previous study, inflammatory components such as COX-2 and interleukins (ILs) have been proven to be significantly associated with this three-hit hypothesis [5].

Another issue brought about by inflammation was oxidative stress (OS), a status wherein there are more reactive oxygen species (ROS) than can be detoxified in the tissues. OS is particularly elevated when macrophages or neutrophils overproduce free radicals [6]. OS can influence cells through DNA point mutation, DNA deletion, DNA rearrangement, or reducing DNA repair. These would alter a cell from its normal cycle and might prevent apoptosis. This process possibly contributes to hyperplasia as well. In real-world practice, chronic inflammation was associated with worse symptoms [7], and OS was significantly decreased 60 days after prostatectomy to BPH [8].

On the basis of the abovementioned role of inflammation and OS in BPH and the current limitation in medicines, this study aims to examine a new derivative with a phenolic structure from a natural extract possessing antioxidative abilities and its effective treatment dosage in controlling BPH. To our knowledge, polyphenolic compounds in fruits, such as apples, could help lower cardiovascular events, reduce the risk of cancer, downregulate inflammation, and prevent metabolic disease. Phloretin, a flavonoid extracted from apple leaves [9], could not only improve the metabolic conditions but also suppress the expression of macrophage and proinflammatory genes [10]. Suppressing the former could interrupt the three-hit process and decrease APC in the prostate, whereas suppressing the latter could downregulate OS in the prostate. Moreover, the ability of phloretin to induce apoptosis, which can attenuate the development of prostate cancer, was also researched [11]. This makes phloretin a possible and attractive derivative to prevent BPH. This study is the first animal model discussing the therapeutic effect of phloretin on BPH.

## 2. Materials and Methods

### 2.1. Animals

Sprague–Dawley rats used in this study were obtained from the National Laboratory Animal Center (Taipei, Taiwan). All procedures using animals were reviewed and approved by the Institutional Animal Care and Use Committee of the National Chung Hsing University (NCHU), and the study protocols were approved by the Committee on Animal Research and Care in NCHU (No. 110046). The rats were placed in sterile cages that were regulated for temperature (22 ± 3 °C), humidity (55 ± 5%), and 12 h day/night cycle conditions. The rats were provided with ad libitum free access to sterilized mouse food and water.

### 2.2. BPH induction and Dosage

The rats were divided into 5 groups of 5 rats each. In the control group, the rats received corn oil/DMSO vehicles (*v*/*v*, 95%/5%) and served as the normal control. In the BPH group, the rats were administered with a daily dose of 10 mg/kg testosterone subcutaneously for 28 days. In the other 3 groups, BPH was induced in rats by administering testosterone 10 mg/kg in olive oil subcutaneously and simultaneous treatment with different doses of phloretin (50 and 100 mg/kg × body weight; phr 50 and phr 100 group, respectively) or finasteride (5 mg/kg × body weight; fina group) per orem, for 28 days. Phloretin was administered orally at 2 doses (50 mg/kg, 100 mg/kg) on the basis of our previous studies [12,13]. At the end of the treatment period, the mice were sacrificed by using carbon dioxide (air displacement rate, 30% of the chamber volume/min). The rats were exposed to 50% CO_2_ until they were unconscious and experienced cardiac arrest. The vital parameters such as prostate weight (PW), prostate weight-to-body weight (BW) ratio (PW/BW), DHT, 5α-R level, expression of ROS, and the inflammatory and apoptotic proteins in the prostate tissue were evaluated.

### 2.3. Prostate Weight

After the rats were sacrificed on day 28, their prostate tissues were excised, rinsed, and weighed immediately after removal. The PW/BW ratio was calculated using the following equation: PW/BW ratio = (prostate weight of each rat from the experimental group/body weight of each rat from the experimental group) × 100.

### 2.4. Histological Examination

After the rats were sacrificed on day 28, their prostate tissue samples were fixed in 4% formalin, dehydrated with a graded alcohol series, embedded in paraffin, and then cut into 4-μm-thick sections. The sections were stained with hematoxylin and eosin (Sigma-Aldrich Inc., St. Louis, MO, USA). The images were captured by using a microscope (Leica, Wetzlar, Germany) under a 10× eyepiece and a 10× objective lens (100×). The epidermal thickness was measured using ImageJ software (National Institutes of Health [NIH], Bethesda, Montgomery County, MD, USA).

### 2.5. Determination of DHT in Serum

The DHT analyses were performed by using the serum from centrifuged blood (4 °C and 10,000 rpm for 5 min) of sacrificed rats on day 28. The levels of DHT were determined by using commercial enzyme-linked immunosorbent assay (ELISA) kits (cat. 11-DHTHU-E01, ALPCO Diagnostics, Salem, NH, USA) and performed according to the manufacturer’s instructions. Results were expressed in pg/mL units.

### 2.6. Assay for Antioxidant Markers in Prostate Tissue

On day 28, the total protein was isolated from 100 mg of prostate tissue by homogenization in 1 mL of a tissue protein extraction reagent (Pierce, Rockford, IL, USA) with protease inhibitors (Roche, Indianapolis, IN, USA). The supernatants obtained after centrifugation of the homogenates (10,000× g for 5 min at 4 °C) were used for protein assays, with the remainder of the supernatant stored at −80 °C for further analysis. The protein concentrations were quantified by a bicinchoninic acid protein assay kit (Thermo Fisher Scientific, Waltham, MA, USA). The prostate concentrations of malondialdehyde (MDA) levels, superoxide dismutase (SOD), and glutathione peroxidase (GSH-px) were determined using commercial kits purchased from the MyBiosource Company (San Diego, CA. USA). All procedures were performed according to the manufacturer′s recommendations.

### 2.7. Assay for Cytokine Expressions in Prostate Tissue

Prostate tissues from rat were collected by the end of day 28. The assay for the levels of IL-6, IL-8, and IL-17A was performed in the prostate tissue of BPH and treated groups using specific ELISA kits (MyBiosource Company, San Diego, CA. USA). All procedures were performed according to the manufacturer′s recommendations.

### 2.8. Immunohistochemistry

After rats were sacrificed on day 28, their prostate tissues were removed and sectioned in the sagittal plane. Then, prostate tissues would be embedded into paraffin blocks to support the tissue structure. The paraffin-embedded slides were de-paraffinized and rehydrated before antigen retrieval. Antigen was retrieved with citrate buffer in a high-pressure cooking pot, followed by quenching of endogenous peroxidase and blocking with normal serum. The slides were incubated overnight at 4 °C with antibodies to the proliferative cell nuclear antigen (PCNA; polyclonal, ab18197, dilution to 1:200; Abcam, Cambridge, UK). Tissue sections were subsequently washed and incubated with HRP-conjugated goat anti-rabbit secondary antibody (polyclonal; 111-035-144; dilution to 1:2000; Jackson ImmunoResearch, West Grove, Chester PA, USA) for overnight at 4 °C. Enzyme activity was then detected by adding 3,3′-diaminobenzidine (DAB) chromogen (LabVision Corp., Fremont, CA, USA). The images were captured by using a microscope (Leica, Wetzlar, Germany) with a 10× eyepiece and a 40× objective lens (400×). Then, 5 randomly distributed fields within the ventral prostate lobe on each slide were analyzed. The cells with brown nuclei were considered PCNA-positive, counted, and expressed as a percentage of the total cells.

### 2.9. Terminal Deoxynucleotidyl Transferase dUTP Nick End Labeling Staining of Prostate Tissue

Prostate tissues from rats were collected by the end of day 28. The paraffin-embedded slides were de-paraffinized and rehydrated. TUNEL staining was performed by using a Click-iT™ TUNEL Colorimetric IHC Detection Kit (Thermo Fisher Scien-tific, Waltham, MA, USA). The images were captured using a microscope (Leica, Wetzlar, Germany) with a 10× eyepiece and a 20× objective lens (200×).

### 2.10. Assay for Cleaved Caspase-3 in Prostate Tissue

Prostate tissues from rats were collected by the end of day 28. The total protein was isolated from 100 mg of prostate tissue by homogenization in 1 mL of a tissue protein extraction reagent (Pierce, Rockford, IL, USA) with protease inhibitors (Roche, Indianapolis, IN, USA). The supernatants were obtained after centrifugation of the homogenates (10,000 *g* for 5 min at 4 °C). The prostate concentrations of the MDA levels were measured by using an active caspase-3 ELISA kit (MBS7244630, MyBioSource).

### 2.11. NF-κB Activity Assay

On day 28, the prostate tissues were harvested by homogenization in 1 mL of a tissue protein extraction reagent (Pierce, Rockford, IL, USA) with protease inhibitors (Roche, Indianapolis, IN, USA), and the nuclear extracts were prepared by using the NE-PER Nuclear and Cytoplasmic Extraction system (Thermo Fisher Scientific, Waltham, MA, USA). For each assay, a total of 10 μg of nuclear extract was used in a TransAM NF-κB p65 ELISA kit (Active Motif, Carlsbad, CA, USA). The procedure was performed according to the manufacturer’s instructions.

### 2.12. Western Blot Analysis

Prostate tissues from rats were collected by the end of day 28. They were homogenized and proteins were extracted with RIPA lysis buffer. Equal amounts (30 μg) of proteins were loaded onto SDS-PAGE gels. Following electrophoresis, the proteins were transferred to nitrocellulose membranes, which were incubated with primary antibodies against 5α-R ((SRD5A1), polycolonal, 1:1000; Thermo Fisher Scientific, Waltham, MA, USA), COX-2 (clone SP21, 1:1000; Thermo Fisher Scientific, Waltham, MA, USA), B-cell lymphoma-2 (Bcl-2) antigen (polycolonal, 1:1000; Thermo Fisher Scientific, Waltham, MA, USA), Bcl-2 associated X protein (Bax) (polycolonal, 1:1000; (Invitrogen; Thermo Fisher Scientific, Inc., Waltham, MA, USA) and anti-GAPDH (polycolonal, 1:5000; Thermo Fisher Scientific, Waltham, MA, USA). Following the additional washing with PBS-T, the membrane was incubated with HRP-conjugated goat anti-rabbit secondary antibody (polyclonal; 111-035-144; dilution to 1:10,000; Jackson ImmunoResearch, West Grove, PA, USA) for overnight at 4 °C, and the immunoreactive bands were detected with an enhanced chemiluminescence (ECL) (GE Healthcare Life Sciences) system and developed by using the Hansor Luminescence Image System (Taichung, Taiwan). All bands in the blots were normalized to the level of GAPDH for each lane. The band density was measured with the ImageJ v1.47 program for Windows from the National Institute of Health (NIH) (Bethesda, Rockville, MD, USA).

### 2.13. Statistical Analyses

Values were presented as standard deviation, and the results were analyzed statistically via one-way analysis of variance followed by Tukey′s multiple comparisons with GraphPad Prism software (version 9.0; GraphPad Software, Inc., San Diego, CA, USA). Type-1 error was set as 5%, and similar results were obtained from 3 independent experiments.

## 3. Results

### 3.1. Phloretin Prevents Hyperplasia in Testosterone-Induced BPH Rats

**Figure 1:**
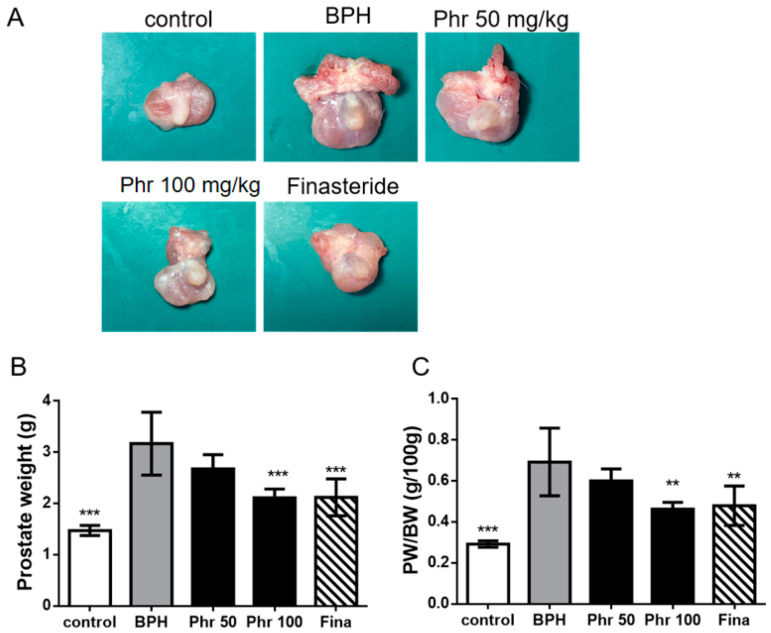
The effect of phloretin on enlarged prostate in testosterone-induced BPH rat model. (**A**) Representative images showing changes of prostatic tissues from each experimental group on day 28. (**B**) Prostate weight (PW) and (**C**) prostate weight to body weight (PW/BW) ratio was measured and analyzed. Prostate weight to body weight (PW/BW) ratio. (**) *p* < 0.01, (***) *p* < 0.001 versus the BPH group, as determined by one-way ANOVA with Tukey’s multiple comparison test.

The mean prostate weight (PW) of rats in the BPH group was significantly increased as compared to the normal rats, indicating that testosterone-induced BPH in rats (Figure 1A,B). The PW was reduced considerably in the phr100 group and fina group rats (Figure 1A,B). Moreover, administration of 100 mg phloretin and finasteride showed efficacy in reducing the PW/BW ratio, as compared to BPH (Figure 1C). However, there was no difference between the phr 50 group and BPH group (Figure 1C) in terms of PW and PW/BW.

### 3.2. Phloretin Suppressed the Hyperplastic Patterns in Prostate Tissue of BPH Rats

**Figure 2:**
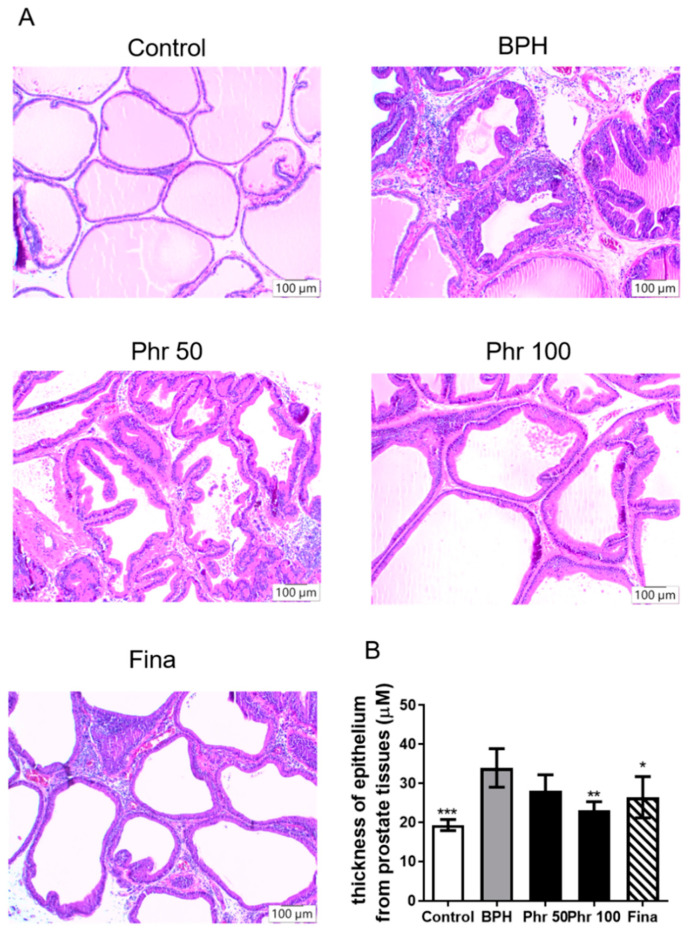
The effects of phorelatin on the hyperplastic patterns in prostate tissue of BPH rats. (**A**) Histological analysis for hematoxylin and eosin-stained prostatic tissues sections from each experimental group on day 28 (original magnification ×100). (**B**) The epithelial thickness of the prostate tissues. (*) *p* < 0.05, (**) *p* < 0.01, (***) *p* < 0.001 versus the BPH group, as determined by one-way ANOVA with Tukey′s multiple comparison test.

Histological examination (Figure 2) of prostate tissue revealed that rats in the BPH group had more glandular hyperplasia with a decreased glandular luminal area (Figure 2A) and increase thickness of the prostatic epithelial cell layer (Figure 2B) than control group. Rats of the phr50 group exhibited moderate suppression of prostate hyperplasia patterns, and rats of phr100 group markedly restored the cellular architecture from testosterone-induced BPH condition. The epithelial cell thickness was also reduced, and the luminal area increased considerably in the phr100 group (Figure 2A,B). There were near normal prostatic glands with mild focal inflammation in the fina group (Figure 2A).

### 3.3. Daily 100 mg/kg Phloretin Significantly Decreased the Serum DHT Level in BPH Rats

**Figure 3:**
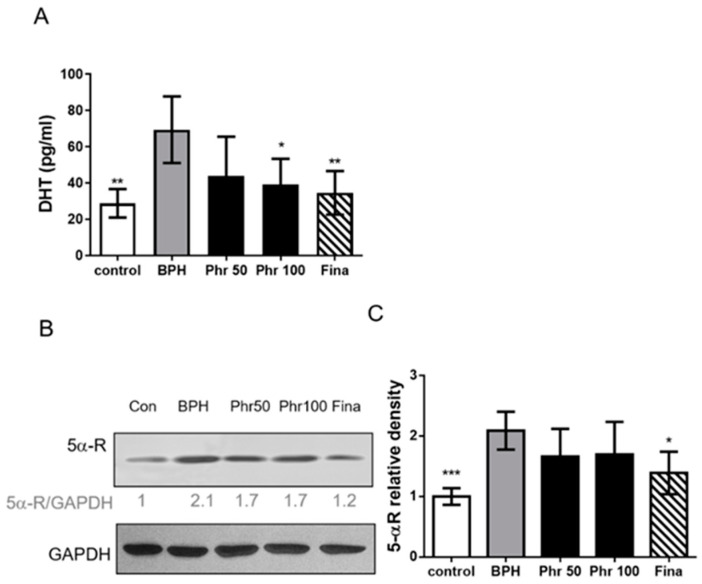
The effects of phloretin on the serum DHT and prostatic 5α-R level in rats of all groups. Serum and prostatic tissues’ homogenates (100 μg/well) were collected on day 28 from each group of rats. (**A**) Serum ELISA for DHT. (**B**) Prostatic tissues were collected on day 28, the protein expression levels of 5α-R were measured using Western blots. (**C**) Densiometric measurements of protein levels were normalized to the corresponding GAPDH protein level using ImageJ software. (*) *p* < 0.05, (**) *p* < 0.01, (***) *p* < 0.001 versus the BPH group, as determined by one-way ANOVA with Tukey’s multiple comparison test.

The levels of serum DHT and prostatic tissue 5α-R were determined via ELISA kits and Western blotting, respectively. Data in Figure 3 indicated that the prostates of rats in BPH group had increased serum DHT (Figure 3A) and tissue 5α-R (Figure 3B,C) levels, compared with the control group. Besides, daily administration of finasteride decreased both the levels of DHT and 5α-R in serum and prostate tissue, respectively. Administration of phloretin 100 mg/kg could significantly decrease the level of serum DHT without affecting prostatic 5α-R (Figure 3A). However, although phloretin of 50 and 100 mg/kg could lower 5α-R, this effect did not reach a statistical difference (Figure 3B,C).

### 3.4. Phloretin Regulated the Expression of Inflammatory Markers in BPH Rats

**Figure 4:**
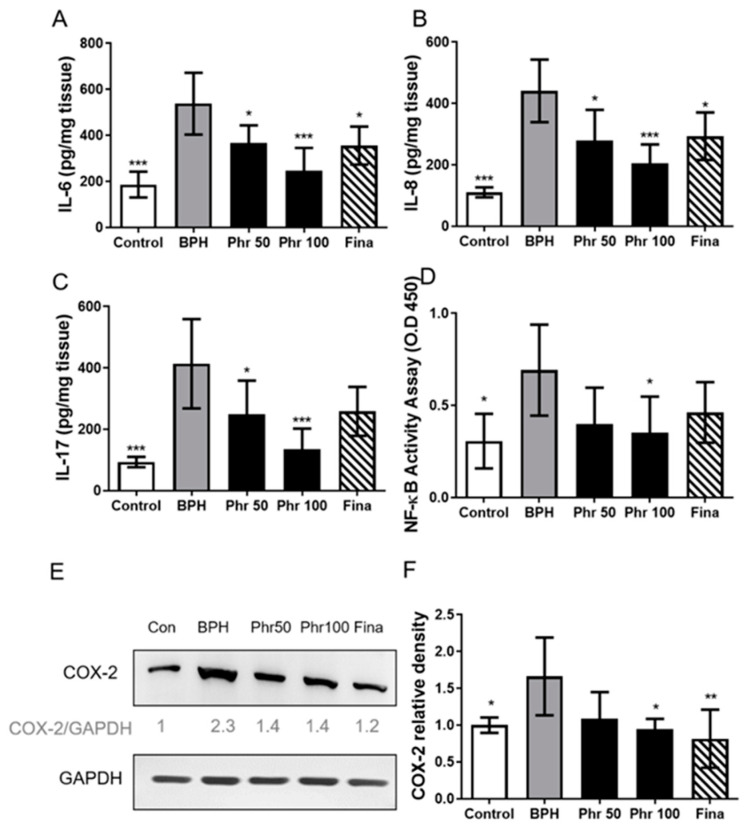
The expression of inflammatory markers in prostate tissue of all rats. Levels of (**A**) IL-6, (**B**) IL-8, (**C**) IL-17 in prostate tissue homogenates. Prostate tissue (100 μg/well) was collected on day 28 from each group of rats for assessing cytokine profiles by ELISA. (**D**)NF-κB p65 DNA-binding activity in nuclear extracts of prostate tissue homogenates was determined by using the TransAM kit, representing the optical density at 450 nm (OD450) values. (**E**) Prostate tissue was collected on day 28, the protein expression levels of COX-2 were measured using Western blots. (**F**) Densiometric measurements of protein levels were normalized to the corresponding GAPDH protein level using ImageJ software (*) *p* < 0.05, (**) *p* < 0.01, (***) *p* < 0.001 versus the BPH group, as determined by one-way ANOVA with Tukey’s multiple comparison test.

As shown in Figure 4, rats in BPH group had more increasing levels in IL-6, IL-8, and IL-17A than the control group (Figure 4A–C) in prostate tissues. Similarly, testosterone treatment (BPH group) could markedly increase the expression of nuclear NF-κB binding activity (Figure 4D) and COX-2 expression (Figure 4E,F) in prostate tissues. Although rats of the phr50, phr100, and fina group had decreasing effects on the abovementioned inflammatory factors (Figure 4A–F), only daily 100 mg/kg phloretin could down-regulate all these factors to a significant level.

### 3.5. Phloretin Inhibited the Expression of PCNA in the Prostate

**Figure 5:**
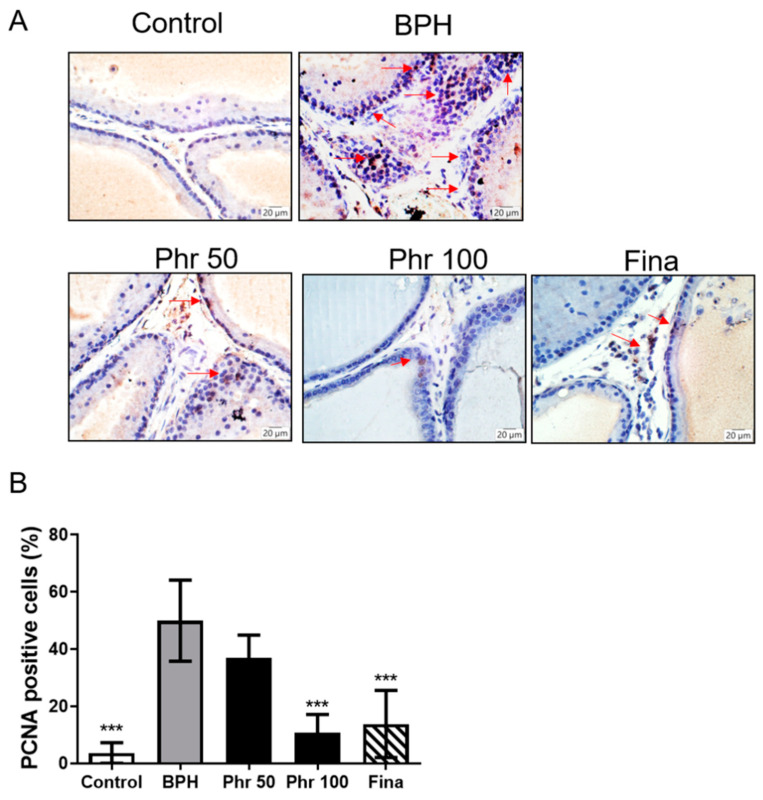
The effect of phloretin on the expression of PCNA in the prostate tissues. (**A**) PCNA staining of prostate epithelium in rats with BPH (day 28). Red arrows indicate the PCNA-positive cells (400×). (**B**)The graph shows the percentages of PCNA-positive cells against the total number of cells. (***) *p* < 0.01, versus the BPH group, as determined by one-way ANOVA with Tukey′s multiple comparison test.

The expression of proliferating cell nuclear antigen (PCNA) protein was elevated in the prostate tissues in the BPH group, compared with the control group. After finasteride treatment, the expression of PCNA was reduced in the fina group, compared with the BPH group (Figure 5A,B). Reduction of expression of PCNA protein was also observed in the rats of phr100 group. Although the proliferative cells were decreased in the phr50 group, this did not achieve a significantly statistical difference (Figure 5A,B).

### 3.6. Effect of Phloretin on Prostatic MDA, SOD, and GSH-Px

The OS and antioxidants results are listed in Supplement Table S1. Testosterone resulted in a significant increase in the level of MDA in the BPH group (13.1 ± 1.9 nmol/mg, control group: 5.1 ± 1.2 nmol/mg; *p* < 0.001), whereas the administration of 100 mg/kg phloretin (phr 100; 6.5 ± 1.6 nmol/mg; *p* < 0.001 versus BPH group) and finasteride (fina group; 8.9 ± 2.2 nmol/mg; *p* < 0.05 versus BPH group) markedly decreased the levels of MDA. A notable decrease in SOD (7.2 ± 1.2 U/mg, control group: 14.4 ± 1.5; *p* < 0.001) and GSH-Px (27.4 ± 4.6 U/g control group: 45.2 ± 5.3 U/g; *p* < 0.001) was observed in the BPH group. The levels of SOD and GSH-Px were significantly elevated in the phr 100 (SOD: 11.84 ± 1.9 U/mg; *p* < 0.01 versus BPH group, GSH-Px: 44.8 ± 7.6 U/g; *p* < 0.01 versus BPH group) and fina groups (SOD: 10.3 ± 1.5 U/mg; *p* < 0.05 versus BPH group, GSH-Px: 38.3 ± 5.7 U/g; *p* < 0.05 versus BPH group) in comparison with the BPH group. Although the phr 50 group had significantly lower levels of MDA (8.9 ± 2.7 nmol/mg; *p* < 0.05 versus BPH group), the elevation of the levels of the antioxidants was not significant. These results indicate that phloretin 100 mg/kg could significantly attenuate the OS in BPH.

### 3.7. Phloretin Regulated the Expression of Apoptosis Markers in BPH Rats

**Figure 6:**
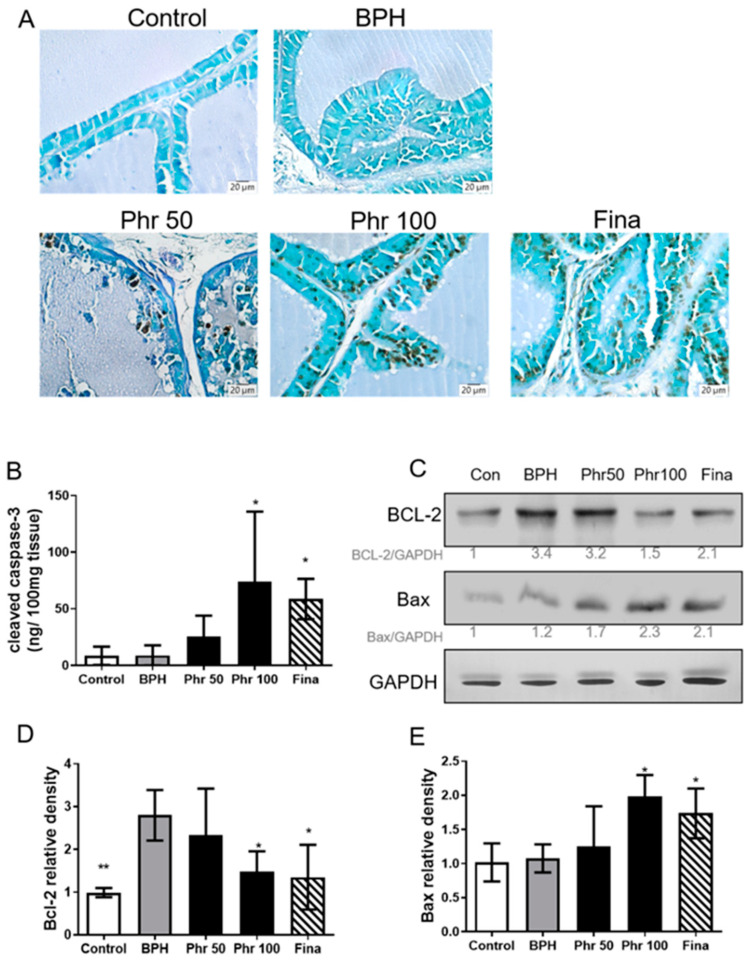
The effects of phorelatin on the expression of apoptosis markers in the prostate of BPH rats. (**A**) Representative photomicrographs of prostate tissue from each experimental group on day 28, showing apoptotic cells, determined by TUNEL staining (magnification, ×200). (**B**) Prostate tissue (100 μg/well) was collected on day 28 from each group of mice for assessing cleaved caspase-3 protein expression by ELISA. The protein expression levels of (**C**) Bcl-2 and (**D**) Bax were measured using Western blots. (**E**) Densiometric measurements of protein levels were normalized to the corresponding GAPDH protein level using ImageJ software. (*) *p* < 0.05, (**) *p* < 0.01 versus the BPH group, as determined by one-way ANOVA with Tukey′s multiple comparison test.

In histological stains of Figure 6, there was no significant difference in the number of TUNEL positive cells between the control group and the BPH group (Figure 6A). In the phr100 group, there was a greatly increased number of TUNEL positive cells compared with the BPH group. Additionally, there was a considerable increase in the cleaved caspase-3 level in the prostate tissues of rats in the phr100 group (Figure 6B). Furthermore, in the phr100 and fina groups, it showed a significant decrease in the anti-apoptotic protein Bcl-2 and an increase in the expression of the pro-apoptotic protein Bax, compared with the BPH group (Figure 6C–E)

## 4. Discussion

From the three-hit process, the prostate cells in BPH could stimulate the secretion of IL-6 and IL-8. Additionally, activating the alloreactive CD4 T cells could further release IL-17, which in turn could amplify the production of IL-6 and IL-8 and form a positive proinflammatory loop [14]. Recently, several bioactive agents that focused on interrupting this loop have been studied, and their results have been promising [15,16]. Earlier, we mentioned that the testosterone-type-2 5α-R-DHT axis could stimulate the growth of stromal and epithelial prostatic cells [4] via autocrine or paracrine signaling, with the inflammatory response as another possible pathway. In our experiment, we observed elevated levels of IL-6, IL-8, and IL-17 in rats with testosterone-induced BPH, which may imply that the effect of inflammation and androgens in stimulating the growth of prostate cells might not be independent of each other and may actually have a common pathway. Our results also showed that phloretin 100 mg/kg/day could successfully attenuate the ILs and DHT to decrease the PW and PW/BW.

NF-κB has long been studied for its role in the proinflammatory pathway. Its importance in the development of prostate cancer was believed to be associated with its anti-apoptotic function [17,18]. Activating the anti-apoptotic gene Bcl-2 could exert its ability to hamper prostate cells from normal decay. Additionally, NF-κB could upregulate the gene-producing IL-6 and possibly stimulate the abovementioned loop, further enhancing the proliferation of prostatic cells [17]. A study also mentioned that in low-dose bisphenol A (BPA)-induced BPH, increased levels of NF-κB were also noted [19]. In our experimental rat models, 100 mg/kg/day of phloretin not only attenuated both NF-κB and Bcl-2 but also exerted its pro-apoptotic function by upregulating the apoptotic gene Bax. Additionally, cleaved caspase-3, which performs apoptosis, was enhanced in our experiment, reflecting the true pro-apoptotic effect of phloretin by upregulating Bax. In the tissue assessment of PCNA and TUNEL, both the reduced effect of the former and the enhanced effect of the latter simultaneously demonstrated the antiproliferative and pro-apoptotic effects of phloretin.

In the inflammatory process, the arachidonic acid (AA) metabolic pathway is mostly involved. Under the effect of prostaglandin (PG) synthases, AA will be degraded into functional PGs, affecting the inflammatory and hormonal processes. Among them, COX-2, a PG H synthase, will be significantly upregulated when induced by various factors such as hormone and inflammation-related signals. In low-dose BPA-induced BPH, COX-2 was associated with the proliferation of prostatic cells, and suppressing it could promote apoptosis and inhibit proliferation [19]. In our experiment, phloretin 100 mg/kg/day could lower the COX-2 level. However, COX-2 is involved not only in the inflammation but also in the protection of the gastrointestinal tract. Although the value of COX inhibitors in helping improve the BOO of BPH in conjunction with alpha-blockers has been studied [20], its clinical application has been restricted by the possible adverse effects on the gastrointestinal tract [21]. If COX inhibitors also affected COX-1, the additional cardiovascular risk must be considered [21]. In terms of cardiovascular risk, phloretin could exert its cardioprotective benefits by inhibiting intracellular chloride channels [22,23]. In terms of its gastrointestinal benefits, phloretin has been proven to attenuate ulcerative colitis in animal models via the downregulation of OS [24], and it also provides hepatoprotection by increasing the levels of antioxidants [25].

To assess OS, MDA, SOD, and GSH-px were studied to examine the function of phloretin. Increased lipid peroxidation will result in the downregulation of the radical-scavenging system, which will worsen the inflammatory condition. In clinical studies, plasma MDA [26] has been shown to be related to inflammatory reactions [27] and even cancer [28]. However, its correlation to prostate cancer and BPH are seldom discussed. In a prospective study [29], several OS-related indices were comprehensively unveiled. MDA was significantly related to prostate cancer; however, its relationship with BPH was not significant. Notably, this study was underpowered and might represent a type-2 error. As for SOD and GSH-px, lower levels were observed in BPH than in normal healthy patients [29]. Between SOD and GSH-px, the role of the latter has been studied more often. In a meta-analysis, GSH-px was related to prostate cancer and BPH development, and its level was lower in prostate cancer than in BPH [30]. Although the conclusion regarding GSH-px and its relationship with prostate cancer and BPH suffered from great heterogeneity, where I^2^ values were 88% and 91%, this might still provide a temporary trend of assessment. In this study, phloretin 100 mg/kg/day could not only decrease MDA but also increase the levels of SOD and GSH-px. This might imply that phloretin could not only prevent BPH but also play a role in preventing prostate cancer.

In the developed countries of Asia, the Western lifestyle was hypothesized to be related to the increasing trend of prostate cancer [31]. One example is a high-fat diet, which was one of the essential contributing factors. This may have caused excessive production of ROS and OS, resulting in a rise in cases of BPH. Similar to our experiment, the impact of a high-fat diet was investigated [32]. It could increase levels of IL-6 and COX-2, thereby promoting the inflammatory process. Its anti-apoptotic ability was observed in increasing NF-κB and Bcl-2 and downregulating Bax. At the same time, the increase in OS was evident from the elevated levels of MDA and decreased levels of SOD and GSH-px. This way, the proposed therapeutic effect of phloretin in this study might antagonize the effects of a Western lifestyle and help ameliorate the increasing trend of prostate cancer and BPH.

**Figure 7:**
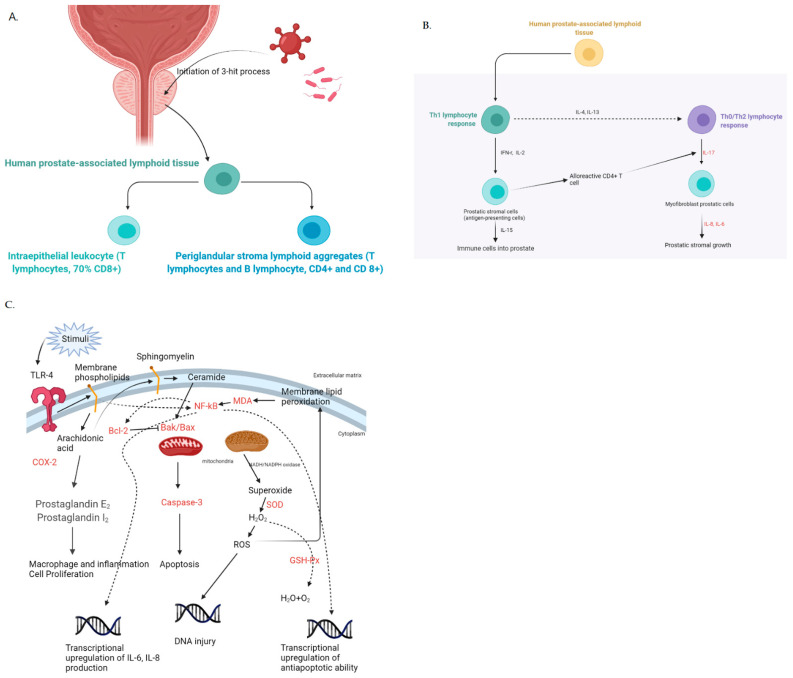
Illustrations regarding the inflammatory hypothesis of prostatic hyperplasia and red-color characters were the examined factors inhibited by phloretin 100 mg/kg/day. (**A**) The initiation of 3-hit process and its subsequent downstream activation. (**B**) After activation of the inflammatory process, the major ILs involved in stimulating hyperplasia were IL-17, IL-6, and IL-8. All these factors were suppressed by phloretin 100 mg/kg/day. (**C**) In cellular aspects of TLR4 overexpression, phloretin 100 mg/kg/day exerted its anti-hyperplasia ability through inhibiting COX-2, upregulating Bax and caspase-3, downregulating Bcl-2, upregulating SOD and GSH-Px, and downregulating MDA and NF-κB.

Our study showed that 100 mg/kg/day of phloretin may prevent BPH by hampering the proinflammatory and anti-apoptotic conditions, promoting apoptotic ability, and alleviating OS (Figure 7). However, only a partial effect was observed when 50 mg/kg/day of phloretin was administered. Except for the downregulated proinflammatory ILs, no pro-apoptotic effect and decreased OS were observed. This partial effect might be attributed either to the type-2 error in our small-size study or to an inferior dosage; an accurate treatment effect for 50 mg/kg/day requires a larger sample size. To date, this is the first animal model that verified the therapeutic effect of 100 mg/kg/day phloretin in preventing BPH. Aside from the alpha-blockers and 5α-R inhibitors, phloretin might serve as a new derivative in treating BPH. The design of its practical application should incorporate side effects such as libido and erection into consideration. Aside from the abovementioned side effects of alpha-blockers and 5α-R inhibitors, the possible gastrointestinal side effects of phloretin, particularly gastritis and gastric ulcer, should be considered since it also possesses the ability to inhibit COX-2, as literature describing them is not yet available. Once the lesser side effects of phloretin have been addressed, its use can be more widespread and may increase the patients′ adherence to medications, thereby improving the quality of life.

## 5. Conclusions

Phloretin, a natural flavonoid, could help prevent BPH in rats by downregulating proinflammatory ILs, inhibiting anti-apoptotic genes, promoting apoptotic activity, and alleviating OS. With an effective treatment dosage of 100 mg/kg/day examined successfully in this study, future experiments could further focus on its cardiovascular and gastrointestinal side effects in the animal model and extend it to human trials if proven safe.

## Data Availability

The data that support the findings of this study are available from the corresponding author upon reasonable request.

## Supplementary Materials

The following are available online at https://www.mdpi.com/article/10.3390/life11080743/s1, Table S1: The effect of phloretin on Prostatic MDA, SOD, and GSH-Px.

## References

[1] Miñana B., Molero J.M., Rolán A.A., Martínez-Fornes M.T., Pinto R.C., Mingot D.L., Carreño Á., Palacios-Moreno J.M. (2021). Real-world therapeutic management and evolution of patients with benign prostatic hyperplasia in primary care and urology in Spain. Int. J. Clin. Pract..

[2] Zhou Z., Cui Y., Wu J., Ding R., Cai T., Gao Z. (2019). Meta-analysis of the efficacy and safety of combination of tamsulosin plus dutasteride compared with tamsulosin monotherapy in treating benign prostatic hyperplasia. BMC Urol..

[3] Ng M., Baradhi K.M. (2020). Benign Prostatic Hyperplasia. StatPearls.

[4] La Vignera S., Condorelli R.A., Russo G.I., Morgia G., Calogero A.E. (2016). Endocrine control of benign prostatic hyperplasia. Andrology.

[5] Vignozzi L., Rastrelli G., Corona G., Gacci M., Forti G., Maggi M. (2014). Benign prostatic hyperplasia: A new metabolic disease?. J. Endocrinol. Investig..

[6] Chughtai B., Lee R., Te A., Kaplan S. (2011). Role of Inflammation in Benign Prostatic Hyperplasia. Rev. Urol..

[7] Nickel J.C., Roehrborn C.G., Castro-Santamaria R., Freedland S.J., Moreira D. (2016). Chronic Prostate Inflammation is Associated with Severity and Progression of Benign Prostatic Hyperplasia, Lower Urinary Tract Symptoms and Risk of Acute Urinary Retention. J. Urol..

[8] Ercan M., Alp H.H., Kocaturk H., Bakan N., Gul M. (2019). Oxidative stress before and after surgery in benign prostatic hyperplasia patients. Andrologia.

[9] Commisso M., Bianconi M., Poletti S., Negri S., Munari F., Ceoldo S., Guzzo F. (2021). Metabolomic Profiling and Antioxidant Activity of Fruits Representing Diverse Apple and Pear Cultivars. Biology.

[10] Alsanea S., Gao M., Liu D. (2017). Phloretin Prevents High-Fat Diet-Induced Obesity and Improves Metabolic Homeostasis. AAPS J..

[11] Kang D., Zuo W., Wu Q., Zhu Q., Liu P. (2020). Inhibition of Specificity Protein 1 Is Involved in Phloretin-Induced Suppression of Prostate Cancer. BioMed Res. Int..

[12] Wang S.-P., Lin S.-C., Li S., Chao Y.-H., Hwang G.-Y., Lin C.-C. (2016). Potent Antiarthritic Properties of Phloretin in Murine Collagen-Induced Arthritis. Evid. Based Complement. Altern. Med..

[13] Wu C.-S., Lin S.-C., Li S., Chiang Y.-C., Bracci N., Lehman C.W., Tang K.-T., Lin C.-C. (2020). Phloretin alleviates dinitrochlorobenzene-induced dermatitis in BALB/c mice. Int. J. Immunopathol. Pharmacol..

[14] Penna G., Fibbi B., Amuchastegui S., Cossetti C., Aquilano F., Laverny G., Gacci M., Crescioli C., Maggi M., Adorini L. (2009). Human Benign Prostatic Hyperplasia Stromal Cells as Inducers and Targets of Chronic Immuno-Mediated Inflammation. J. Immunol..

[15] Penna G., Fibbi B., Amuchastegui S., Corsiero E., Laverny G., Silvestrini E., Chavalmane A., Morelli A., Sarchielli E., Vannelli G.B. (2009). The vitamin D receptor agonist elocalcitol inhibits IL-8-dependent benign prostatic hyperplasia stromal cell proliferation and inflammatory response by targeting the RhoA/Rho kinase and NF-kB pathways. Prostate.

[16] Elbaz E.M., Amin H.A., Kamel A.S., Ibrahim S.M., Helmy H.S. (2020). Immunomodulatory effect of diallyl sulfide on experimentally-induced benign prostate hyperplasia via the suppression of CD4+T/IL-17 and TGF-β1/ERK pathways. Inflammopharmacology.

[17] Suh J., Rabson A.B. (2004). NF-κB activation in human prostate cancer: Important mediator or epiphenomenon?. J. Cell. Biochem..

[18] Chappell W.H., Candido S., Abrams S.L., Russo S., Ove R., Martelli A.M., Cocco L., Ramazzotti G., Cervello M., Montalto G. (2018). Roles of p53, NF-κB and the androgen receptor in controlling NGAL expression in prostate cancer cell lines. Adv. Biol. Regul..

[19] Wu S., Huang D., Su X., Yan H., Ma A., Li L., Wu J., Sun Z. (2020). The prostaglandin synthases, COX-2 and L-PGDS, mediate prostate hyperplasia induced by low-dose bisphenol A. Sci. Rep..

[20] Gokkaya C.S., Aktas B.K., Ozden C., Bulut S., Karabakan M., Erkmen A.E., Memis A. (2015). Flurbiprofen alone and in combination with alfuzosin for the management of lower urinary tract symptoms. Central Eur. J. Urol..

[21] Juszczak K., Drewa T. (2015). The cardiovascular and gastrointestinal adverse effects of cyclooxygenase inhibitors seems to be a major concern that restricts their use in the treatment of urinary bladder dysfunction. Central Eur. J. Urol..

[22] Wang X., Takahashi N., Uramoto H., Okada Y. (2005). Chloride Channel Inhibition Prevents ROSdependentApoptosis Induced by Ischemia-Reperfusion in Mouse Cardiomyocytes. Cell. Physiol. Biochem..

[23] Malekova L., Tomaskova J., Novakova M., Stefanik P., Kopacek J., Lakatos B., Pastorekova S., Krizanova O., Breier A., Ondrias K. (2007). Inhibitory effect of DIDS, NPPB, and phloretin on intracellular chloride channels. Pflugers Arch..

[24] Zhang Z., Li S., Cao H., Shen P., Liu J., Fu Y., Cao Y., Zhang N. (2019). The protective role of phloretin against dextran sulfate sodium-induced ulcerative colitis in mice. Food Funct..

[25] Ren D., Liu Y., Zhao Y., Yang X. (2016). Hepatotoxicity and endothelial dysfunction induced by high choline diet and the protective effects of phloretin in mice. Food Chem. Toxicol..

[26] Nielsen F., Mikkelsen B.B., Nielsen J.B., Andersen H.R., Grandjean P. (1997). Plasma malondialdehyde as biomarker for oxidative stress: Reference interval and effects of life-style factors. Clin. Chem..

[27] Cherian D.A., Peter T., Narayanan A., Madhavan S.S., Achammada S., Vynat G.P. (2019). Malondialdehyde as a Marker of Oxidative Stress in Periodontitis Patients. J. Pharm. Bioallied Sci..

[28] Kangari P., Farahany T.Z., Golchin A., Ebadollahzadeh S., Salmaninejad A., Mahboob S.A., Nourazarian A. (2018). Enzymatic antioxidant and lipid peroxidation evaluation in the newly diagnosed breast cancer patients in Iran. Asian Pac. J. Cancer Prev..

[29] Kaya E., Ozgok Y., Zor M., Eken A., Bedir S., Erdem O., Ebiloglu T., Ergin G. (2017). Oxidative stress parameters in patients with prostate cancer, benign prostatic hyperplasia and asymptomatic inflammatory prostatitis: A prospective controlled study. Adv. Clin. Exp. Med..

[30] Sajjaboontawee N., Supasitthumrong T., Tunvirachaisakul C., Nantachai K., Snabboon T., Reiche E.M.V., Simão A.N.C., Maes M. (2020). Lower thiol, glutathione, and glutathione peroxidase levels in prostate cancer: A meta-analysis study. Aging Male.

[31] Kimura T., Egawa S. (2018). Epidemiology of prostate cancer in Asian countries. Int. J. Urol..

[32] Li Y., Shi B., Dong F., Zhu X., Liu B., Liu Y. (2019). Effects of inflammatory responses, apoptosis, and STAT3/NF-κB- and Nrf2-mediated oxidative stress on benign prostatic hyperplasia induced by a high-fat diet. Aging.

