# Supplementary material for: Phloretin Ameliorates Testosterone-Induced Benign Prostatic Hyperplasia in Rats by Regulating the Inflammatory Response, Oxidative Stress and Apoptosis
Source: Life (Basel). 2021 Jul 26;11(8):743. doi: 10.3390/life11080743 (PMC8399389; doi:10.3390/life11080743)
Supplement: Supplementary file 1 [file life-11-00743-s001.zip › life-1296014-supplementary.pdf]

life

Supplementary Materials

Phloretin Ameliorates Testosterone-Induced Benign Prostatic Hyperplasia in Rats by Regulating the Inflammatory Response, Oxidative Stress and Apoptosis

Chao Yu Hsu [1,2], Yi Sheng Lin [1], Wei Chun Weng [1,3], Lauren Panny [4], Hsiang Lai Chen [1,2], Min Che Tung [1], Yen Chuan Ou [1,*], Chi Chien Lin [5,6,7,8,9,*] and Che Hsueh Yang [1,*]

1 Division of Urology, Department of Surgery, Tungs' Taichung MetroHarbor Hospital, Taichung 435, Taiwan; t4361@ms.sltung.com.tw (C.Y.H.); t12197@ms.sltung.com.tw (Y.S.L.); t1142@ms.sltung.com.tw (M.C.T.); t10527@ms.sltung.com.tw (W.C.W.); t3811@ms.sltung.com.tw (H.L.C.)
2 PhD Program in Translational Medicine, Rong Hsing Research Center for Transitional Medicine, National Chung Hsing University, Taichung 402, Taiwan
3 Department of Nursing, Jen-Teh Junior College of Medicine, Nursing and Management, Miaoli 356, Taiwan
4 Department of Biomedical Sciences and Pathobiology, Virginia-Maryland College of Veterinary Medi-cine, Virginia Polytechnic Institute and State University, Blacksburg, VA 24061, USA; laurenpanny@vt.edu
5 Institute of Biomedical Science, The iEGG and Animal Biotechnology Center, National Chung-Hsing University, Taichung 402, Taiwan
6 Department of Biotechnology, Asia University, Taichung 413, Taiwan
7 Department of Medical Research, China Medical University Hospital, China Medical University, Taichung 406, Taiwan
8 Department of Medical Research, Taichung Veterans General Hospital, Taichung 407, Taiwan
9 Department of Pharmacology, College of Medicine, Kaohsiung Medical University, Kaohsiung 807, Taiwan
* Correspondence: t10669@ms.sltung.com.tw (Y.C.O.); lincc@email.nchu.edu.tw (C.C.L.); b101098093@tmu.edu.tw (C.H.Y.)

Table S1. The effect of phloretin on Prostatic MDA, SOD, and GSH-Px. Prostate tissue homogenates (100 μg/well) were collected on day 28 from each group of rat.In oxidative stress assessment, the decreased oxidative marker, MDA, and elevated antioxidants, SOD, were seen in Phr 100 group. Meanwhile, GSH-Px, a cytosolic enzyme catalyzing peroxide radicals, was observed elevated in Phr 100 group. (*) $p < 0.05$, (**) $p < 0.01$, (***) $p < 0.001$ versus the BPH group, as determined by one-way ANOVA with Tukey's multiple comparison test.

	Dose (mg/kg/day)	MDA (nmol/mg)	SOD (U/mg)	GSH-Px (U/g)
Control	-	5.1±1.2***	14.4±1.5***	45.2±5.3***
BPH	-	13.1±1.9	7.2±1.2	27.4±4.6
Phr 50	50	8.9±2.7*	10.1±1.5	33.6±7.3
Phr 100	100	6.5±1.6***	11.4±1.9**	44.8±7.6**
Fina	3	8.9±2.2*	10.3±1.5*	38.3±5.7*
